# Association of systemic immune-inflammatory index with in-stent restenosis in patients with and without diabetes mellitus

**DOI:** 10.3389/fcvm.2025.1419314

**Published:** 2025-01-20

**Authors:** Xunwei Deng, Qiaoting Deng, Qunji Zhang, Jingyuan Hou

**Affiliations:** ^1^ Institute of Cardiovascular Disease, Meizhou People’s Hospital (Huangtang Hospital), Meizhou, China; ^2^ Guangdong Provincial Engineering and Technology Research Center for Molecular Diagnostics of Cardiovascular Diseases, Meizhou People's Hospital (Huangtang Hospital), Meizhou, China; ^3^ Meizhou Academy of Medical Sciences, Meizhou People's Hospital (Huangtang Hospital), Meizhou, China

**Keywords:** systemic immune-inflammatory index, in-stent restenosis, diabetes mellitus, restricted cubic spline, nonlinear relationship

## Abstract

**Background:**

Systemic inflammation plays a vital role in the pathogenesis and prognosis of cardiovascular disease (CAD). The systemic immune-inflammation index (SII) has been developed as a cost-effective and practical predictor for CAD outcomes. This study aimed to determine the association between the SII and the risk of ISR among ACS patients with and without diabetes mellitus (DM).

**Methods:**

In this retrospective cohort study, a total of 1,652 patients who underwent percutaneous coronary intervention (PCI) from February 2015 to December 2020 and were finally enrolled after follow-up with coronary angiography. The SII was calculated based on neutrophil, platelet and lymphocyte counts. Multivariable logistic regression models were employed to assess the associations between SII and ISR prevalence. Additionally, the interaction test and subgroup analysis were performed to evaluate the robustness of our findings. Furthermore, restricted cubic splines analysis was applied to visualize the relationship between the SII and the risk of ISR. Employing Spearman's rank correlation analysis to investigate the relationship between SII levels and the time to ISR occurrence.

**Results:**

In the whole cohort enrolled in this study, 128 (7.7%) participants developed angiographic evidence of ISR. The results demonstrated that the SII level significantly increased in patients with ISR compared to those with non-ISR, and these findings were similar in patients with and without DM. After adjusting for confounders, the multivariate logistic regression analysis revealed that participants with higher SII levels had a significantly increased risk of ISR for diabetics (all *P* < 0.05), and this significant association was observed in patients with more severe ISR (triple-coronary artery lesions). Additionally, RCS analysis reveals that there is a J-shaped nonlinear correlation between SII and ISR in the entire study cohort with (*P* for overall <0.001, and *P* for nonlinearity = 0.0058, respectively). Moreover, a threshold effect can be observed in the entire cohort, with an inflection point at the log2-SII value of 9.276 (SII = 620). Specifically, increased SII was linearly associated with ISR in diabetics (*P* for overall = 0.0007 and *P* for nonlinearity = 0.4316, respectively), indicating that the correlation between SII and ISR is stronger in diabetic patients than in those without diabetes. Spearman's rank correlation analysis demonstrated that elevated SII levels are related to earlier ISR onset in diabetics (*r* = −0.272, *P* = 0.049).

**Conclusion:**

Our study suggests that SII may be an affordable and convenient marker that could be applied to predict the risk of ISR among ACS patients. Moreover, the study emphasized that high SII is an independent predictor of more severe and earlier ISR and may be helpful for patients' risk stratification, especially those with comorbid DM.

## 1 Introduction

Coronary artery disease (CAD) remains a leading cause of morbidity and mortality worldwide and accounts for nearly 30% of cardiovascular disease global burden (1). Invasive procedures like percutaneous coronary intervention (PCI) coupled with stent insertion are the prevailing initial treatment for patients with acute coronary syndrome (ASC) (2, 3). Despite advancements in coronary stent technology, in-stent restenosis (ISR) remains a noticeable clinical challenge, leading to serious cardiovascular complications and adverse outcomes (4). The discovery of biomarkers for early identification of ISR may be helpful in clinical decisions and treatment.

Preceding studies have revealed that the pathogenesis of ISR involves a complicated interaction of cellular and molecular factors, including inflammation, smooth muscle cell proliferation, and endothelial dysfunction (5, 6). Accumulating evidence indicated that blood cells, specifically neutrophils, monocytes, and platelets, are implicated in systemic inflammation and are tied to the causative factors of CAD (7, 8) and peripheral arterial disease (9). In the intricate pathogenesis of ISR, the inflammatory orchestration involving neutrophils, lymphocytes, and platelets unfolds dynamically. In the context of acute coronary incidents, neutrophils are instrumental in inflicting injury upon the endothelium and in the aggregation of platelets (10, 11). Simultaneously, lymphocytes, representing the immune modulatory response, influence the prognosis of ISR (12). Platelets, essential in ISR and neointimal hyperplasia, are implicated in systemic infections, inflammation, and thrombotic states (13).

The systemic immune-inflammation index (SII), conceptualized by Hu et al. in 2014, has emerged as a valuable biomarker reflecting the intricate interplay between the systemic immune response and inflammation (14). Widely studied in the context of cancer prognosis, the SII has shown its versatility in predicting outcomes for various malignancies (15, 16). Notably, recent research has expanded the application of SII to cardiovascular studies. Emerging evidence has indicated a significant association between elevated SII values and adverse cardiovascular events, including coronary artery lesions, acute coronary syndromes, and major cardiovascular events, surpassing the predictive power of traditional risk factors (17–19). The SII's ability to integrate peripheral platelet, neutrophil, and lymphocyte count is a comprehensive gauge of the host's status in terms of inflammation and immunity, making it a potentially superior tool in cardiovascular disease research.

Diabetes mellitus (DM) stands as a well-established common complication and risk factor for CAD, often exacerbating one another (20). Individuals with DM frequently face a heightened burden of CAD, leading to increased rates of rehospitalization and adverse outcomes (21). A pivotal feature in the pathophysiology of type 2 diabetes mellitus (T2DM) is chronic tissue inflammation, which drives the recruitment, accumulation, and activation of neutrophils and lymphocytes (22). Recent studies underscore that diabetic patients experience cardiovascular advantages from antidiabetic agents, which are due not only to the reduction of blood glucose levels but also partly to the anti-inflammatory effects of these medications. For example, preclinical and clinical studies of SGLT2 inhibitors have demonstrated such effects (23–25). A thorough literature review was conducted using databases such as PubMed, Embase, and Cochrane Library, with search terms including “Systemic Immune-Inflammation Index”, “in-stent restenosis”, and “diabetes mellitus”. While there have been a few scattered reports on the correlation between SII and ISR (26, 27), no studies have provided evidence regarding the correlation between SII and ISR in the context of diabetes. Accordingly, we purpose to analyze the relationship between SII and ISR in patients diagnosed with ACS, encompassing those with diabetes and those without.

## 2 Materials and methods

### 2.1 Study design and population

**Figure 1:**
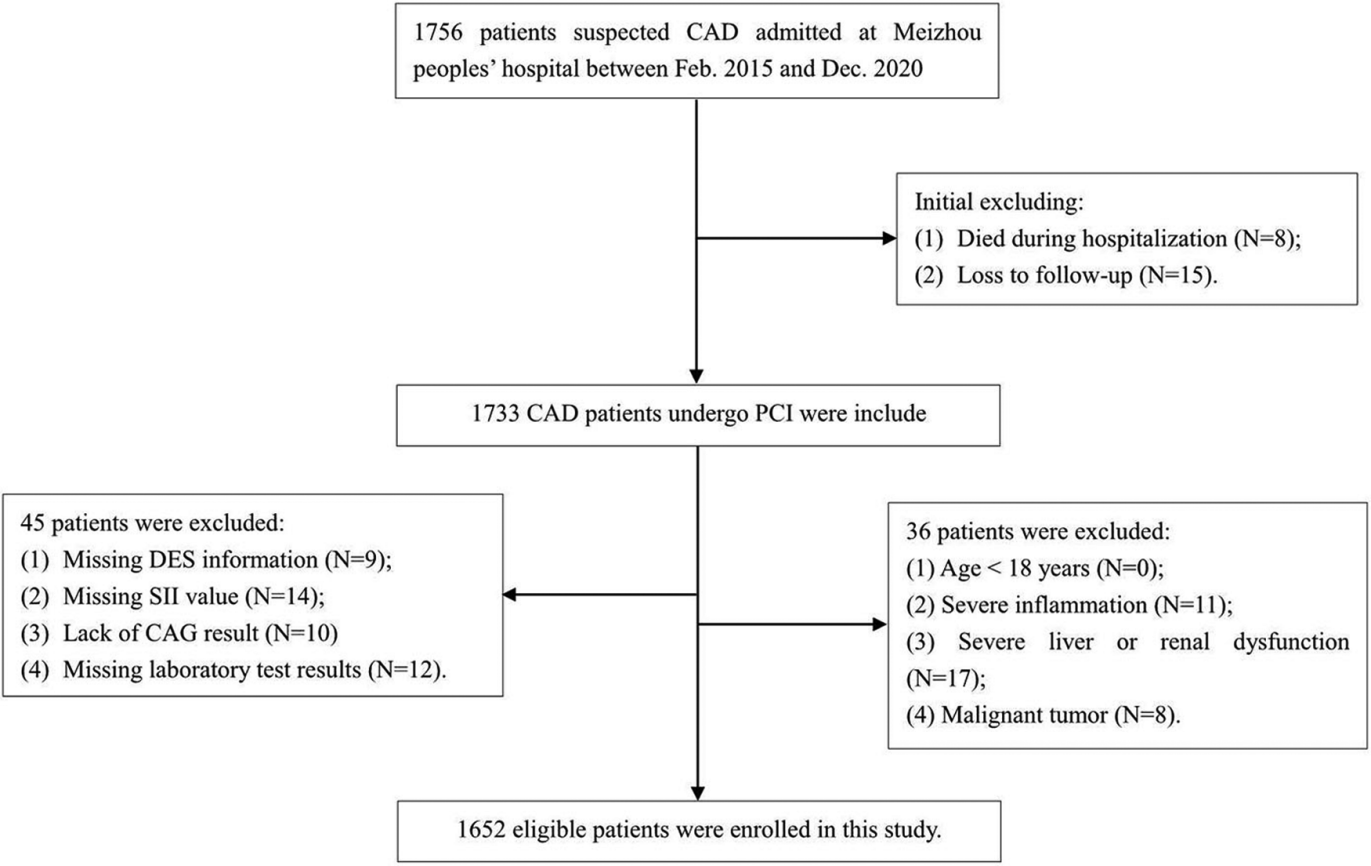
Flowchart of patient inclusion and exclusion for this study.

Consecutive patients who were admitted for acute coronary syndrome (ACS) in Meizhou People's Hospital, and underwent follow-up angiography ranging from 3 to 24 months after receiving coronary drug-eluting stents (DES) implantation for the first time, were retrospectively enrolled from February 2015 to December 2020. The diagnosis of ACS was made in line with the criteria specified in the guidelines (28). The protocol of this study adhered to the principles of the Declaration of Helsinki and was authorized by the ethics committee of the institution. Written informed consent was not required as the study was retrospective, and all participant records were anonymized and ensure confidentiality. Participants were selected based on the following requirements: (1) patients with acute coronary syndromes; (2) those receiving DES for the first time at our facility; (3) DES was performed according to the Chinese Guidelines for Percutaneous Coronary Intervention (2016) (29); (4) confirmation of intra-stent restenosis (ISR) via coronary angiography during follow-up; (5) relevant demographic characteristics, laboratory and imaging data are complete and available from the hospital information system. Participants excluded from the studies were those aged below 18 years, individuals with incomplete coronary angiography results, insufficient follow-up data, missing Systemic Immune-Inflammation Index (SII) results, severe hepatic or renal dysfunction, patients with acute or chronic inflammatory diseases, autoimmune diseases, and those with malignancies. The detailed screening process is illustrated in Figure 1. A total of 1652 eligible participants ranging from 26 to 92 years were included in the final cohort, all of which were assessed with 2-year follow-up by angiography after DES implantation. Two cardiologists, each operating autonomously, were charged with analyzing the follow-up angiography results and subsequently assigning the participants to either the group with Intra-Stent Restenosis (ISR) or the group without ISR. Furthermore, we investigated the association between SII levels and ISR across diverse subgroups of ACS patients, categorized based on the degrees of coronary artery lesions: specifically, those with single-coronary artery lesion (*n* = 177), double-coronary artery lesions (*n* = 303), and triple-coronary artery lesions (*n* = 1,172). Parallel analyses were conducted within distinct subgroups according to diabetes, comprising 522 diabetics and 1,130 non-diabetics.

### 2.2 Definition

The SII was calculated as platelet count × neutrophil-to-lymphocyte ratio. ISR is characterized by luminal narrowing exceeding 50% within the stent segment or within 5 mm proximal or distal to the stent edges, as confirmed by coronary angiography. Hypertension is indicated by blood pressure measurements ≥140/90 mmHg or antihypertensive therapy; diabetes mellitus is diagnosed by elevated fasting blood glucose or random blood glucose levels, or reception of hypoglycemic drugs; dyslipidemia is characterized by elevated lipid levels or lipid-lowering medication use.

### 2.3 Angiographic analysis and stent implantation

Coronary angiography by radial or femoral artery access using the standard Judkins technique. Intervention and stent implantation in coronary arteries were performed under current practice guidelines (30). The stents used were second-generation DES, which included zotarolimus-eluting and domestic everolimus-eluting stents. Coronary angiographic analysis were managed by experienced interventional cardiologists. All patients were treated with a standardized PCI strategy. Moreover, oral aspirin (600 mg) combined with clopidogrel (300 mg) was administered before emergency PCI. Oral aspirin (100 mg/day) combined with clopidogrel (75 mg/day), or ticagrelor (90 mg twice daily) after emergency PCI and elective PCI were given to the patient for >1 year. During this procedure, the patient was given heparin anticoagulation (100 IU/kg) to maintain the activated coagulation time of 250–350 s. Patients undergo repeat coronary angiography approximately one year after PCI to determine whether ISR has occurred. Early coronary angiography may be considered for patients experiencing postoperative chest tightness and chest pain.

### 2.4 Data collection

Patient demographic information, echocardiography data, past medical history, laboratory parameters, and discharge medication used at the first PCI were collected. All laboratory data were collected for the first time after hospital admission. Fasting peripheral blood samples were gathered following an overnight fast of >8 h to test blood routine and biochemical variables, consisting of blood routine parameters, C-reactive protein (CRP), alanine aminotransferase (ALT), aspartate aminotransferase (AST), blood urea nitrogen (BUN), creatinine (Cr), uric acid (UA), glucose, lipid parameters of total cholesterol (TC), triglyceride (TG). The blood samples were evaluated within 2 h. The coronary angiography characteristics and the stents included lesion vessels, number and length of stents were also collected. All information was collected from the electronic medical recording system of Meizhou People's Hospital by trained physicians who were unaware of the study's intent.

### 2.5 Statistical analysis

**Figure 2:**
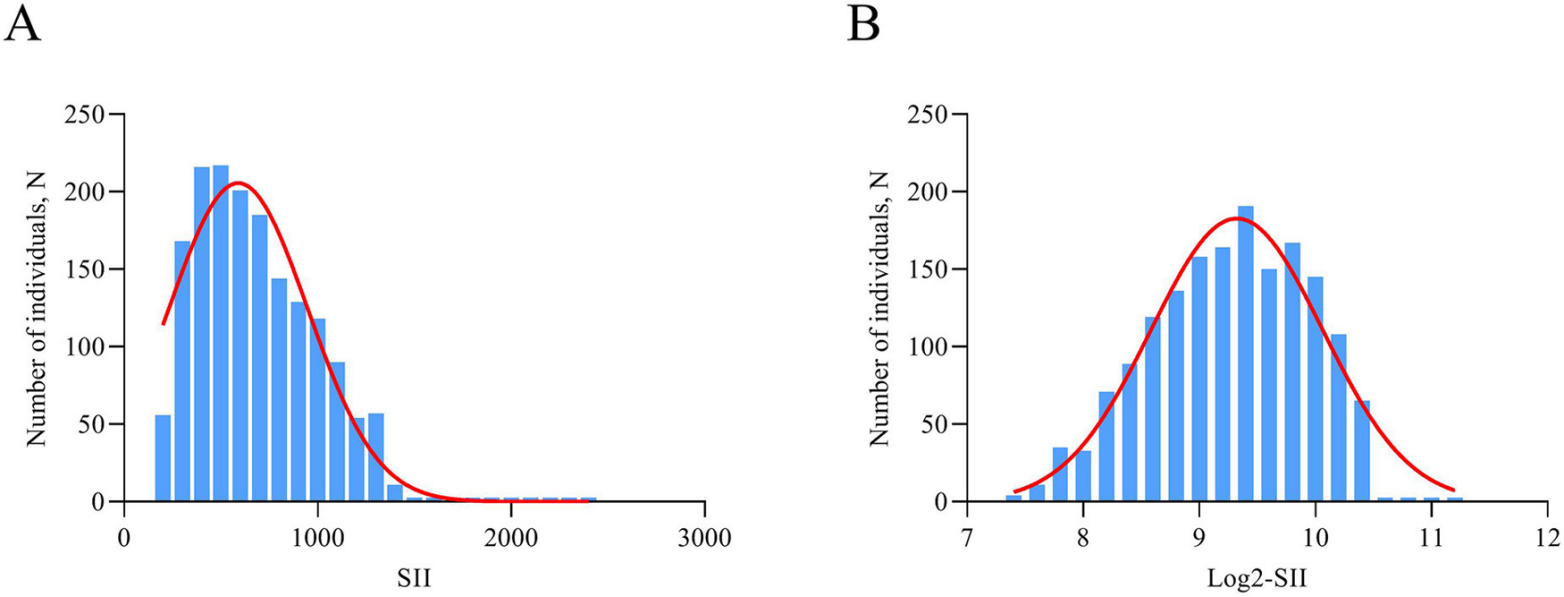
The distribution of SII **(A)** the distribution of log2-transformed SII **(B)**.

Continuous variables were described as mean ± standard deviation (SD) or median [interquartile range (IQR)], and compared by *t*-tests or Wilcoxon rank-sum tests, when appropriate. Categorical variables were presented as frequencies (percentages) and were tested using the χ^2^ test. SII was logarithmic transform when performing analyses because the data was unevenly distributed and skewed to the right (Figure 2). Multivariate logistic regression analysis was performed to assess the relationship between log2-SII and ISR, based on patients' varying degrees of coronary artery lesions and whether or not they had diabetes mellitus. In model 1, covariates were not adjusted. In model 2, traditional risk factors of ISR contained age, gender, and smoking status were adjusted. Model 3 was adjusted for DES stent number, LVEF, lesions of LCX, lesions of RCA, diabetes mellitus, hyperlipidemia, previous MI, usage of ACE inhibitor/ARB, which significantly correlated with ISR in univariate logistic regression analysis, and covariates in Model 2. Furthermore, log 2-SII was analyzed as categorical in multivariate logistic regression according to tertile. Additionally, restricted cubic spline (RCS) analysis was assessed to visualize any potential nonlinear association between log 2-SII and ISR. Finally, stratification and interaction analyses were further carried out to evaluate the heterogeneity of the association in different subgroups. Stratification factors included gender (male/female), age (<60/≥60 years), smoking status (yes/no), LVEF (≥50%/<50%), lesions of LCX (yes/no), and DES stent number (<2/≥2). The relationship between SII and time to ISR occurrence collected over 2 years was evaluated using Spearman's rank correlation analysis. Statistical analyses were conducted utilizing IBM SPSS 21.0, GraphPad Prism 9.0 and R v4.0.3 software (“ggplot2”, “rms” packages; https://www.R-project.org). All statistical tests were two-sided, and a *P*-value less than 0.05 was deemed to indicate statistical significance.

## 3 Results

### 3.1 Baseline characteristics

**Figure 3:**
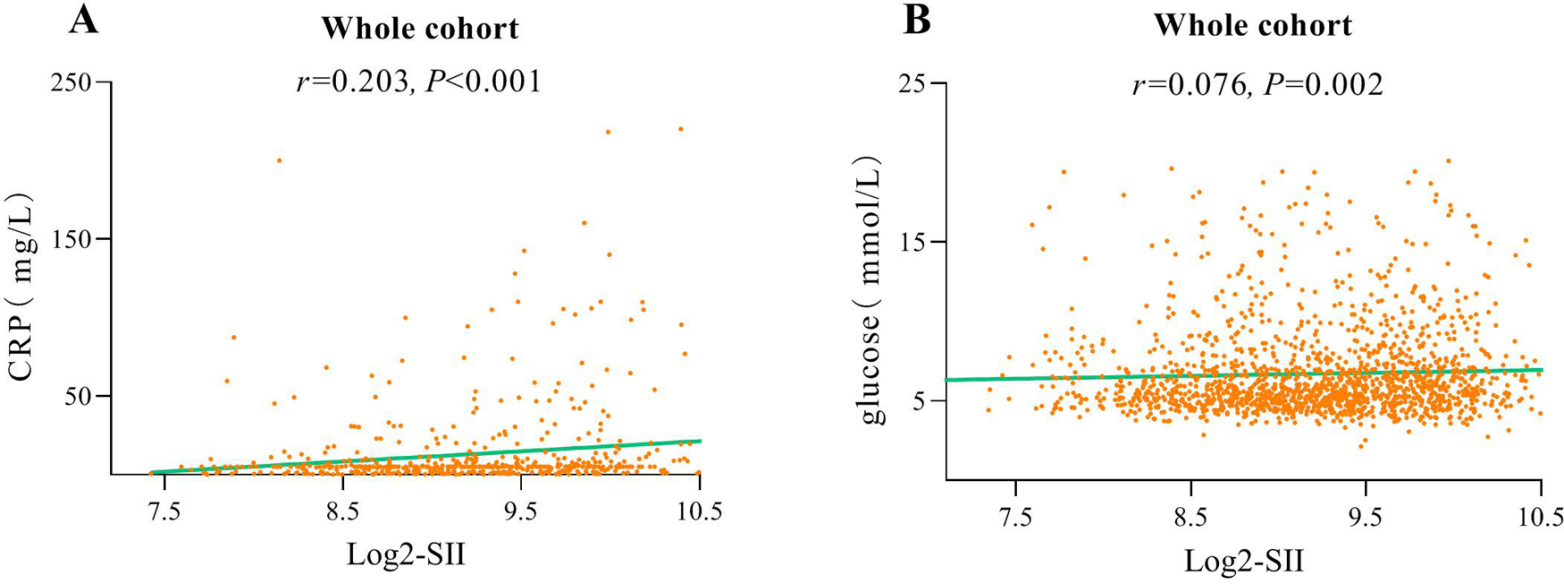
Correlation graph between SII and C-reactive protein **(A)**. Correlation graph between SII and glucose **(B)**.

**Figure 4:**
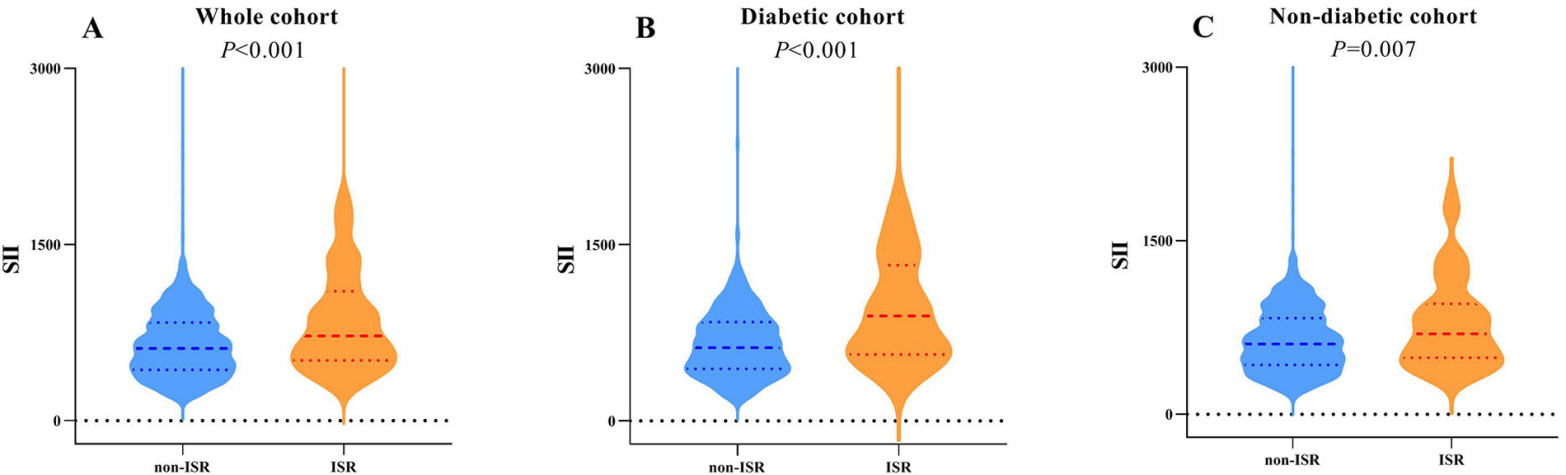
Violin plots of the SII showing the distribution in the non-ISR and ISR groups of the whole cohort **(A)**, diabetic **(B)** and non-diabetic cohorts **(C)** the SII of the ISR group was higher than that of the non-ISR group in all above three cohorts (A, *P* < 0.001; B, *P* < 0.001; C, *P* = 0.007, respectively).

The flowchart of participant selection is shown in Figure 1. First, we excluded participants who died during hospitalization or were lost to follow-up. Participants were further disqualified if they met the exclusion criteria. Overall, a total of 1,652 eligible CAD patients undergoing PCI were involved in this study, of whom 75.2% were male and 65.5% were over 60 years of age, with a median SII of 621.1 (435.8, 849.8). As a practical indicator of systemic immune-inflammatory status, the SII level in this study cohort was positively correlated with the levels of traditional inflammatory markers, such as C-reactive protein (*r* = 0.203, *P* < 0.001), as depicted in Figure 3A. In addition, the SII level was positively correlated with the glucose level (*r* = 0.076, *P* = 0.002), as depicted in Figure 3B. The median follow-up time was 2.0 years, and an ISR prevalence of 7.75% within two years. The variations in baseline characteristics are summarized in Table 1. The results indicated that patients with ISR showed a significantly higher proportion of LCX and RCA lesions (*P* = 0.004 and *P* = 0.031, respectively), more drug-eluting stents implanted in number and length, and higher SII level (all *P* < 0.001). In addition, the ISR cohort showed a significantly lower LVEF level (*P* = 0.011) and lower rates of ACE inhibitor/ARB usage (*P* = 0.023). In terms of complications, the ISR group had a significantly higher prevalence of diabetes and previous MI (all *P* < 0.05), while hyperlipidemia was significantly less than the non-ISR group (*P* = 0.007). While there were no pronounced differences were observed between the ISR and non-ISR groups in terms of demographic information, the levels of laboratory indices except SII and creatinine, and the discharge medication usage besides the ACE inhibitor/ARB. Moreover, the violin plot visually presented that patients in the ISR group had significantly higher SII values than those with non-ISR both in the overall cohort, in diabetic and in non-diabetic patients (all *P* < 0.01), as shown in Figure 4.

**Table 1:** Baseline demographic and clinical characteristics of participants.

Characteristics	Non-ISR (*n* = 1,524)	ISR (*n* = 128)	*P*-value
Age (year), *n* (%)	63.0 (56.0, 71.0)	64.0 (57.0, 71.0)	0.432
Male, *n* (%)	1,143 (75.0)	99 (77.3)	0.555
Systolic blood pressure (mmHg)	137.6 ± 23.4	136.8 ± 25.2	0.740
Diastolic blood pressure (mmHg)	81.9 ± 14.0	82.0 ± 13.9	0.948
Smoking, *n* (%)	482 (31.6)	43 (33.6)	0.646
LVEF (%)	61.0 (53.0, 64.0)	60.0 (43.5, 64.0)	**0.011**
Culprit vessel			
LM, *n* (%)	216 (14.2)	24 (18.8)	0.158
LAD, *n* (%)	1,479 (97.0)	126 (98.4)	0.364
LCX, *n* (%)	1,166 (76.5)	112 (87.5)	**0.004**
RCA, *n* (%)	1,270 (83.3)	116 (90.6)	**0.031**
DES stent number, *n*	1 (1, 2)	1 (1, 2)	**<0.001**
DES stent length, mm	31.0 (23.0, 48.0)	46.0 (29.0, 62.0)	**<0.001**
Cardiovascular history and comorbidities			
Hypertension, *n* (%)	846 (55.5)	67 (52.3)	0.489
Diabetes Mellitus, *n* (%)	469 (30.8)	53 (41.4)	**0.013**
Hyperlipidemia, *n* (%)	525 (34.4)	29 (22.7)	**0.007**
Previous MI, *n* (%)	24 (1.6)	6 (4.7)	**0.011**
Laboratory test			
SII	614.3 (432.6, 835.8)	721.6 (512.7, 1,101.5)	**<0.001**
ALT, U/L	31.0 (20.0, 50.0)	32.0 (19.0, 45.0)	0.679
AST, U/L	32.0 (22.0, 96.8)	31.0 (21.0, 78.5)	0.454
Total cholesterol, mmol/L	5.0 (4.2, 5.8)	4.7 (4.2, 5.5)	0.053
Triglyceride, mmol/L	1.6 (1.1, 2.4)	1.4 (1.0, 2.1)	0.057
RLP-C, mmol/L	0.7 (0.4, 1.1)	0.8 (0.6, 1.1)	0.094
UA, umol/L	365.5 (303.1, 441.0)	369.2 (299.1, 429.6)	0.491
Cr, umol/L	99.0 (87.0, 113.0)	95.0 (81.0, 110.0)	**0** **.** **029**
BUN, mmol/L	5.6 (4.5, 6.8)	5.8 (4.8, 6.7)	0.164
Glucose, mmol/L	5.8 (5.0, 7.3)	6.0 (5.0, 7.5)	0.648
CRP, mg/L	5.0 (2.6, 8.1)	5.8 (1.8, 24.2)	0.055
Discharge medication use			
Statin, *n* (%)	1,491 (97.8)	127 (99.2)	0.289
Aspirin, *n* (%)	1,473 (96.7)	124 (96.9)	0.893
ACE inhibitor/ARB, *n* (%)	1,343 (88.1)	104 (81.3)	**0.023**
Beta-blocker, *n* (%)	1,303 (85.5)	109 (85.2)	0.916
Clopidogrel/Ticagrelor	1,456 (95.5)	120 (93.8)	0.354
Calcium channel blocker, *n* (%)	298 (19.6)	20 (15.6)	0.279

ISR, in-stent restenosis; LVEF, left ventricular ejection fraction; LM, left main; LAD, left anterior descending; LCX, left circumflex; RCA, right coronary artery; DES, drug-eluting stent; MI, myocardial infarction; SII, systemic immune-inflammation index, ALT, alanine aminotransferase; AST, aspartate aminotransferase; RLP-C, remnant-like particle cholesterol; UA, uric acid; Cr, creatinine; BUN, blood urea nitrogen.

Bold values represent *P* ≤ 0.05.

### 3.2 A greater SII level relate to more severe ISR

The results of the multivariate logistic regression analysis of the incidence of log2-SII and ISR in patients with different degrees of coronary artery lesion are listed in Table 2. With the adjustment of other elements that might complicate the interpretation, the results revealed that log2-SII levels were independently related to ISR in all three models for those undergoing double-coronary artery lesions (all *P* < 0.005) and triple-coronary artery lesions (all *P* < 0.001). For patients with single-coronary artery lesions, there were no significant associations between log2-SII and ISR in all models. To further verify this correlation, we converted log2-SII from a continuous variable to a categorical variable (tertiles) for sensitivity analysis, and the results were similar to the primary analysis. Notably, in contrast to the results of continuous variables, elevated levels of log2-SII divided by tertiles were not positively associated with ISR in patients with double-coronary artery lesions (all *P* for trend >0.05). However, the ORs for log2-SII T3 showed a significantly increasing trend of ISR than T1 in all models in those with triple-coronary artery lesions (all *P* for trend <0.05). As in the fully adjusted model, the OR for log2-SII T3 was 1.898 (*P* = 0.017), and the trend *P*-value was 0.016, implying that a greater SII level is significantly linked to more severe ISR, especially for patients with triple-coronary artery lesions.

**Table 2:** Association of log2-SII with ISR in multivariable logistic regression models in patients with different degrees of CAD.

Variable	Model 1			Model 2			Model 3		
	OR (95% CI)	*P*-value	*P* for trend	OR 95% CI)	*P*-value	*P* for trend	OR (95% CI)	*P*-value	*P* for trend
Single-coronary artery lesion									
Log2-SII	1.817 (0.427–7.723)	0.419		2.725 (0.537–13.837)	0.227		1.257 (0.162–9.763)	0.827	
Log2-SII tertiles									
T1	Ref		0.742	Ref		0.459	Ref		0.493
T2	1.032 (0.141–7.558)	0.975		1.273 (0.158–10.276)	0.821		0.532 (0.027–10.435)	0.678	
T3	1.422 (0.193–10.474)	0.730		2.293 (0.283–18.605)	0.437		0.275 (0.007–11.022)	0.493	
Double-coronary artery lesions									
Log2-SII	2.584 (1.010–6.613)	**0** **.** **048**		2.806 (1.051–7.490)	**0** **.** **039**		3.373 (1.134–10.037)	**0** **.** **029**	
Log2-SII tertiles									
T1	Ref		0.086	Ref		0.066	Ref		0.071
T2	1.321 (0.289–6.044)	0.720		1.354 (0.292–6.276)	0.699		1.403 (0.281–6.991)	0.680	
T3	3.254 (0.815–12.991)	0.095		3.660 (0.897–14.933)	0.071		3.937 (0.876–17.686)	0.074	
Triple-coronary artery lesions									
Log2-SII	2.165 (1.595–2.938)	**<0** **.** **001**		2.141 (1.576–2.909)	**<0** **.** **001**		1.925 (1.405–2.639)	**<0** **.** **001**	
Log2-SII tertiles									
T1	Ref		**0** **.** **003**	Ref		**0** **.** **003**	Ref		**0** **.** **016**
T2	1.477 (0.850–2.566)	0.167		1.450 (0.833–2.525)	0.189		1.360 (0.768–2.407)	0.292	
T3	2.169 (1.300–3.619)	**0** **.** **003**		2.143 (1.283–3.580)	**0** **.** **004**		1.898 (1.119–3.219)	**0** **.** **017**	

Model 1 adjusted for none.

Model 2 adjusted for age, gender, and smoking.

Model 3 consisted of DES stent number, LVEF, Lesions of LCX, Lesions of RCA, Diabetes Mellitus, Hyperlipidemia, Previous MI, usage of ACE inhibitor/ARB, and model 2.

SII, systemic immune-inflammation index; ISR, in-stent restenosis; OR, odds ratio; CI, conﬁdence interval; DES, drug-eluting stent; LVEF, left ventricular ejection fraction, LCX, left circumflex, RCA, right coronary artery; MI, myocardial infarction.

Bold values represent *P* ≤ 0.05.

### 3.3 Association of log2-SII with ISR in patients with and without diabetes mellitus

After performing multivariate logistic regression models, the relationship between log2-SII and ISR in patients with diabetes mellitus was investigated. As presented in Table 3, the analysis illustrated that log2-SII was independently risk for developing ISR in both diabetic and non-diabetic patients in the crude model (OR = 2.977, *P* < 0.001 for diabetic, and OR = 1.847, *P* = 0.001 for non-diabetic, respectively). In addition, the positive association between log2-SII and ISR remained stable in the fully adjusted model (OR = 2.321, *P* = 0.001 for diabetic, and OR = 1.770, *P* = 0.003 for non-diabetic, respectively). Moreover, this association showed a trend of increasing odds of ISR with increasing log2-SII tertiles (all *P* for trend <0.05) in the sensitivity analysis of diabetic patients but not non-diabetic patients (*P* for trend = 0.053 in model 3). In the fully adjusted model, compared to the log2-SII in T1, the OR for the incidence of ISR increased in T2 (OR 1.968, 95% CI 0.792–4.889), and climbed to 2.468 (95% CI, 1.028–5.927) for T3 in the diabetic patient.

**Table 3:** Association of log2-SII with ISR in multivariable logistic regression models in patients with and without diabetes mellitus.

Variable	Model 1			Model 2			Model 3		
	OR (95% CI)	*P*-value	*P* for trend	OR (95% CI)	*P*-value	*P* for trend	OR (95% CI)	*P*-value	*P* for trend
Diabetic									
Log2-SII	2.977 (1.866–4.752)	**<0** **.** **001**		2.935 (1.824–4.721)	**<0** **.** **001**		2.321 (1.394–3.864)	**0** **.** **001**	
Log2-SII tertiles									
T1	Ref		**0** **.** **005**	Ref		**0** **.** **005**	Ref		**0** **.** **045**
T2	2.221 (0.938–5.260)	0.070		2.101 (0.882–5.005)	0.206		1.968 (0.792–4.889)	0.145	
T3	3.207 (1.413–7.278)	**0** **.** **005**		3.201 (1.404–7.296)	**0** **.** **006**		2.468 (1.028–5.927)	**0** **.** **043**	
Non-diabetic									
Log2-SII	1.847 (1.275–2.676)	**0** **.** **001**		1.849 (1.275–2.681)	**0** **.** **001**		1.770 (1.214–2.581)	**0** **.** **003**	
Log2-SII tertiles									
T1	Ref		**0** **.** **024**	Ref		**0** **.** **025**	Ref		0.053
T2	1.088 (0.580–2.042)	0.792		1.097 (0.584–2.064)	0.773		1.124 (0.591–2.139)	0.721	
T3	1.918 (1.082–3.398)	**0** **.** **026**		1.914 (1.079–3.394)	**0** **.** **026**		1.770 (0.985–3.182)	0.056	

Model 1 adjusted for none.

Model 2 adjusted for age, gender, and smoking.

Model 3 consisted of DES stent number, LVEF, Lesions of LCX, Lesions of RCA, Diabetes Mellitus, Hyperlipidemia, Previous MI, usage of ACE inhibitor/ARB, and model 2.

SII, systemic immune-inflammation index; ISR, in-stent restenosis; OR, odds ratio; CI, conﬁdence interval; DES, drug-eluting stent; LVEF, left ventricular ejection fraction, LCX, left circumflex, RCA, right coronary artery; MI, myocardial infarction.

Bold values represent *P* ≤ 0.05.

### 3.4 The subgroup analysis and interaction test

**Figure 5:**
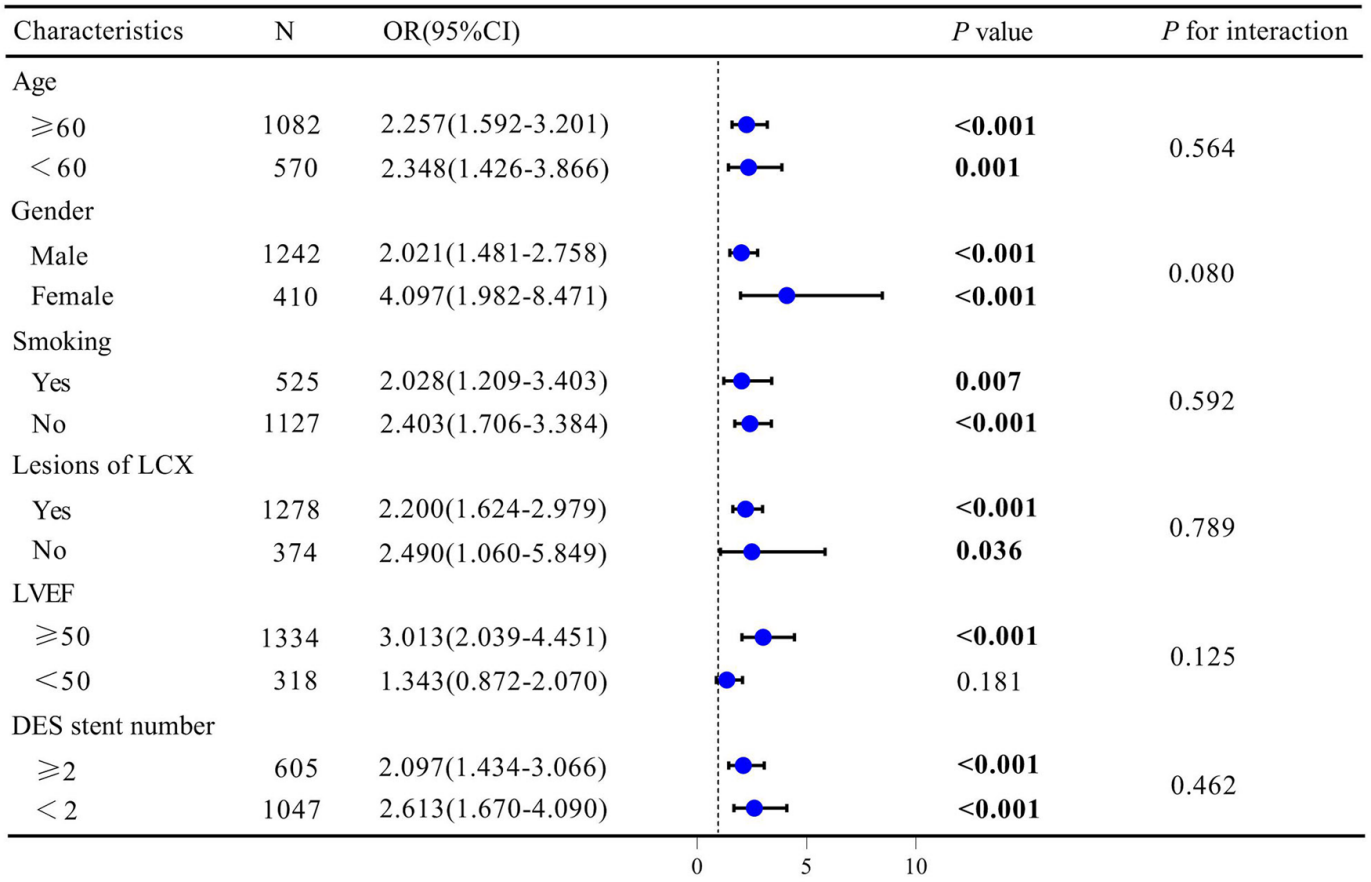
Subgroup analysis for the association between SII and ISR. Odds ratios were calculated based on Log2-SII scores increased by 1. Each stratum was adjusted for age, gender, smoking status, DES stent number, LVEF, Lesions of LCX, Lesions of RCA, Diabetes Mellitus, Hyperlipidemia, Previous MI, and usage of ACE inhibitor/ARB.

Subgroup analysis were carried out to investigate the relationships between the log2-SII and ISR in the entire study cohort. As listed in Figure 5, the results revealed that there were inconsistent relationships between log2-SII level and ISR. Overall, the risk of ISR was not consistently associated with increased log2-SII levels in subgroups stratified by LVEF. In the above subgroups, statistically significant connections between higher log2-SII levels and higher ISR prevalence were mainly among patients with LVEF ≥50% (*P* < 0.001), with ORs ranging from 2.039 to 4.451. Furthermore, the interaction test revealed no significant difference among each stratification between ISR and log2-SII level, with all *P* for interaction exceeding 0.05, demonstrating that there was no significant dependence of these factors on this positive correlation.

### 3.5 Analysis of restricted cubic spline regression

**Figure 6:**
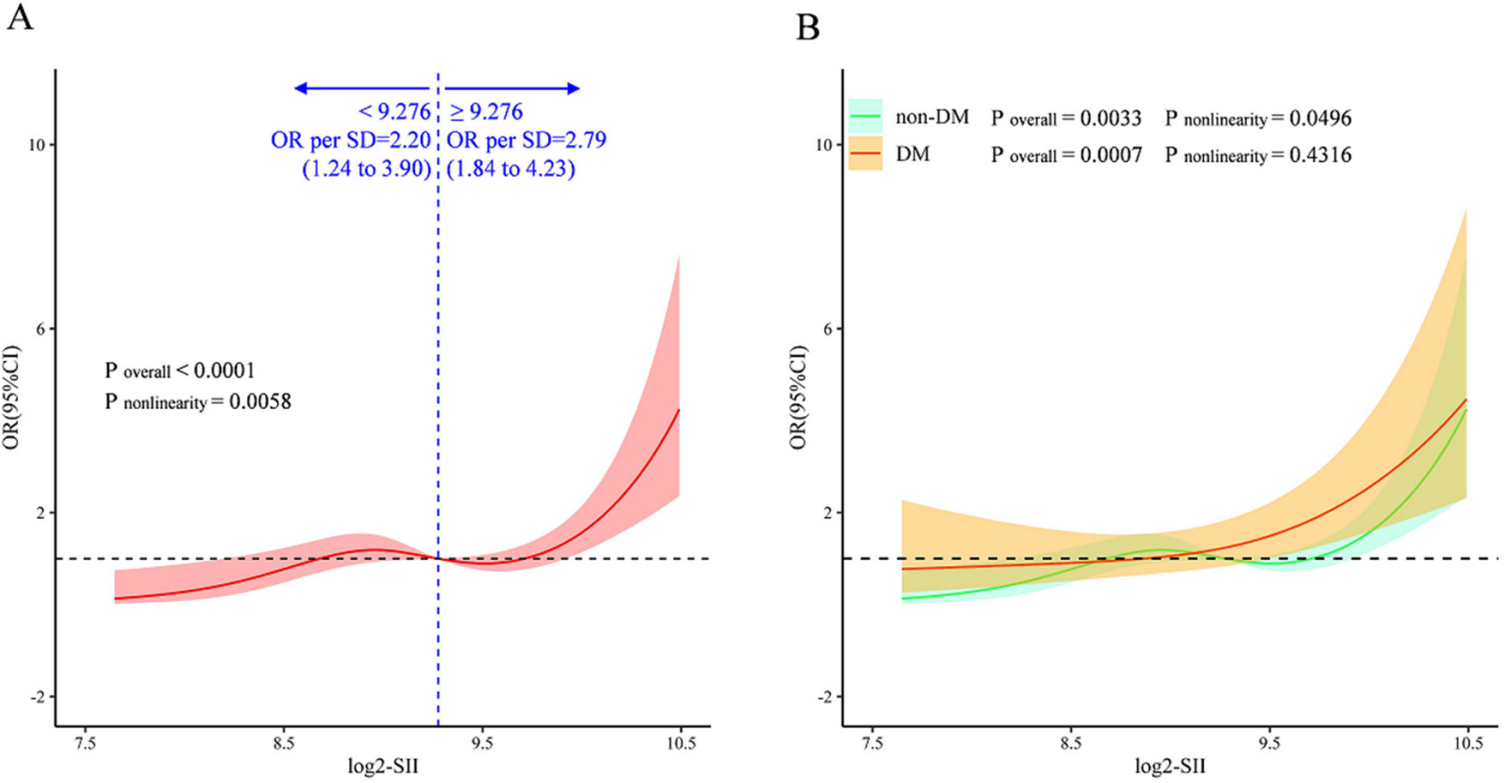
Multivariate RCS regression analysis for the nonlinear association between the SII and ISR in the whole cohort **(A)**, and in the diabetic and nondiabetic cohorts **(B)** ORs (solid lines) and 95% confidence levels (shaded areas) were adjusted for age, gender, smoking status, DES stent number, LVEF, lesions of LCX, lesions of RCA, diabetes Mellitus, hyperlipidemia, previous MI, and usage of ACE inhibitor/ARB. The vertical dashed line indicates the minimum threshold for beneficial association with an estimated OR = 1. OR, odds ratio; CI, confidence interval.

A multivariate restricted cubic splines (RCS) analysis was conducted to visualize and determine whether there was a potential linear or nonlinear association between the log2-SII and ISR in the overall cohort (Figure 6A) and in those with and without diabetes mellitus (Figure 6B). Using RCS, a J-shaped association between log2-SII and ISR was presented, a significant nonlinear connection between log2-SII and ISR risk was discovered in the entire cohort (*P* for nonlinearity <0.001) and non-diabetic participants (*P* for nonlinearity = 0.0033). In addition, a threshold effect can be observed in the entire cohort, with an inflection point at the log2-SII value of 9.276 (SII = 620). Before log2-SII exceeds 9.276, the risk of ISR remains gradually leveled off or even decreases as log2-SII continues to increase (OR per SD = 2.20). Whereas, after passing the inflection point, the risk increases rapidly and the odds ratio per standard deviation higher risk of ISR was 2.79 (1.84–4.23). Moreover, as displayed in Figure 6B, the log2-SII was proved to have a significant positive and almost linear relationship with the probability of the risk of ISR in diabetic participants of this study according to the RCS (*P* for overall = 0.0007 and *P* for nonlinearity = 0.4316, respectively). This finding indicates that the correlation between log2-SII and ISR is both more pronounced and linearly robust in diabetic patients compared to non-diabetic.

### 3.6 Analysis of the correlation between SII levels and the time to ISR onset

**Figure 7:**
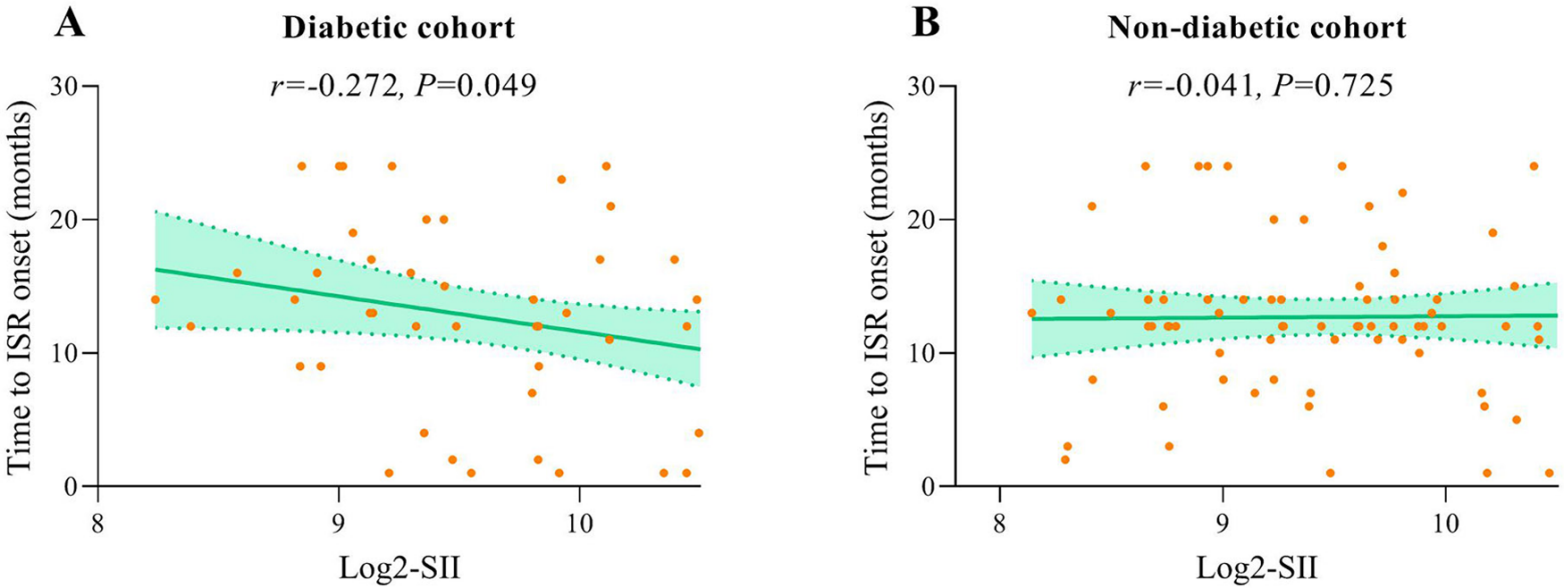
Correlation graphs between SII and time to ISR onset in diabetic **(A)** and non-diabetic cohort **(B)**.

In patients with ISR, the interval from the implantation of a coronary stent to the occurrence of an ISR event within two years was recorded to assess the potential correlation between SII levels and the timing of ISR. Spearman's rank correlation analysis demonstrated a significant negative correlation between SII levels and the time to ISR in diabetic patients, suggesting that elevated SII levels are related to earlier ISR onset (*r* = −0.202, *P* = 0.049). Conversely, no significant correlation was identified between SII levels and the time to ISR in non-diabetic patients (Figure 7).

## 4 Discussion

As far as we can ascertain, our study pioneers the investigation of the correlation between SII levels and ISR within the context of patients with and without DM. The major findings of this research are as follows: (1) participants with ISR had significantly higher SII levels compared to non-ISR; (2) elevated SII levels were independent risk for developing ISR in diabetics; (3) after multivariable adjustment, increased SII values are indicative of a greater risk for ISR, especially for patients with triple-coronary artery lesions; (4) a significant nonlinear association between SII and ISR in non-diabetic, and an almost linear relationship with the probability of the risk of ISR in diabetic participants was further uncovered in the RCS analyses; and (5) elevated SII levels are related to earlier ISR onset in diabetics.

The systemic immune-inflammatory index (SII), integrating neutrophils, lymphocytes, and platelets, serves as a pivotal biomarker in various medical domains. By reflecting both local immune response and systemic inflammation, SII offers a comprehensive assessment of cardiovascular risk, shedding light on potential mechanisms underlying atherosclerosis, thrombosis, and ischemic events (31–33). Particularly, SII has emerged as a significant indicator, offering insights into the intricate interplay between inflammation and cardiovascular diseases (CVDs) (34–37). Recent studies have underscored SII's utility in predicting adverse cardiovascular events post-acute myocardial infarction (AMI) and its association with coronary artery disease (CAD) progression. In a cohort study that enrolled 1,197 AMI patients, Li et al. demonstrated that SII is a more reliable biomarker than platelet-to-lymphocyte ratio (PLR) or neutrophil-to-lymphocyte ratio (NLR) for identifying AMI patients at high-risk of in-hospital MACEs (38). In patients with STEMI, the elevation of SII was not only observed but also linked with a higher risk of malignant ventricular arrhythmias (39). Additionally, elevated SII in patients with atrial fibrillation is an independent predictor of recurrence after the first catheter ablation (36). These discoveries underscore the SII's potential as a noninvasive biomarker, offering valuable insights into the link between systemic inflammation and cardiovascular outcomes. Corresponding to earlier findings, we found that a higher SII level remained an independent risk of developing ISR in diabetics after multivariable adjustment. Based on our findings, in clinical practice, diabetic patients with SII levels exceeding 620 should be given sufficient attention, given their high likelihood of experiencing ISR. The current study investigated the correlation between SII levels and ISR in a larger cohort than the study by Xie et al. (26) and further explored this relationship in patients with ISR with and without diabetes.

The RCS analyses further indicated a nonlinear correlation between SII and ISR in our entire cohort, and after adjusting for confounding factors, a J-shaped relationship was observed (Figure 6A). Interestingly, there is an obvious inflection point in the RCS plot, showing different correlation trends on both sides. As is well known that, the propensity for stent failure and in-stent restenosis is attributed to an inflammatory process that triggers the entry of immune cells and the consequent expansion of smooth muscle cells (40, 41). Elevated blood cells, including platelet, and neutrophil counts may represent a hyper-responsive predisposition to the acute inflammatory stimulus of stenting (42). Prior research has established that the systemic immune-inflammation index (SII) is a biomarker indicative of systemic inflammation. In essence, SII levels provide a metric for gauging the severity of coronary lesions. The J-shaped relationship underscores the association between inflammatory responses and the spectrum of in-stent restenosis (ISR) severity. This pattern is further substantiated by our studies (Table 2). Specifically, we have observed that elevated SII values are predictive of an increased risk of ISR, particularly in patients with triple-vessel coronary artery disease, which represents a more severe form of coronary artery disease.

It is interesting that in the RCS analyses, there is a significant linear relationship between SII and the risk of ISR in diabetic participants (*P* for overall = 0.0007 and *P* for nonlinearity = 0.4316, respectively). Numerous studies have illustrated that DM was an independent risk factor of ISR. There is a synergistic effect between the two that exacerbates the condition, resulting in diabetic individuals undergoing coronary revascularization being more likely to have suboptimal outcomes compared to non-diabetic patients. Abnormal glucose metabolism in diabetics often leads to insulin resistance, which causes vascular fibrosis and affects the structure and function of vascular endothelial cells (43, 44). Endothelial dysfunction is a proatherogenic state, that produces a local inflammatory response that accelerates the proliferation of smooth muscle cells and inflammatory cells, which promotes proliferation of the coronary intima (45–47). Besides, thrombosis is a major comorbidity of T2DM, individuals with T2DM and subclinical inflammation stimulate clotting and activate platelets, and promote the development of ISR (13, 48). Our study further revealed a close relationship between the degree of inflammation *in vivo* and ISR risk. This suggests that anti-inflammatory therapy may confer benefits to these high-risk group. It's worth mentioning that the latest evidence shows that in patients with T2DM, sodium-glucose co-transporter2 inhibitors therapy has a connection with lower rates of ISR independently (49). Researchers have speculated that the protective effect of SGLT2 inhibitors (SGLT2i) against restenosis may not be solely attributed to their hypoglycemic action. Although the precise mechanisms are yet unclear, there is a hypothesis that their anti-inflammatory properties could be contributory. Nevertheless, the study did not specifically evaluate inflammatory markers, necessitating further investigation to clarify this potential pathway. Anti-inflammatory therapies have shown promise in mitigating cardiovascular risks by targeting inflammatory pathways. The study by Bona et al. focuses on inflammation as a possible therapeutic target of ACS and discusses various anti-inflammatory therapies aimed at mitigating ACS, emphasizing their potential to reduce vascular inflammation and subsequent complications like ISR (50). Similarly, Biasucci et al. (51) explored the promises and challenges of targeting inflammation in the post-CANTOS trial era, suggesting that these interventions not only aim to reduce the acute inflammatory response but also potentially attenuate chronic inflammation implicated in the progression of vascular diseases including ISR. In light of these findings, the potential role of inflammation as a therapeutic target in managing cardiovascular diseases becomes paramount. The association between inflammation, quantified by the SII, and ISR is indeed evident from our study findings. Future research is warranted to further investigate whether the SII can serve as a useful biomarker for evaluating the therapeutic efficacy of ISR.

Limitations are inherent in this study. Initially, the typical issue with retrospective research poses a challenge in eliminating the potential for selection bias and the influence of unaccounted confounding variables. Secondly, as a single-center study, the generalisability of these findings to a wider population still needs to be further demonstrated externally. Thirdly, the current study only examined the correlation between baseline SII measurements at admission and ISR, and it is unclear whether changes in SII levels during follow-up affect current outcomes. Finally, the exclusion of participants who died during hospitalization could introduce selection bias, as those who died may have had different baseline characteristics, comorbidities, or more severe conditions that could influence the SII and the risk of ISR. Consequently, the findings of our study may not fully represent the entire population of patients undergoing PCI, particularly those at the highest risk of adverse outcomes. Future studies should consider including these patients to adjust for this potential bias to better understand the full spectrum of risk factors associated with ISR.

## 5 Conclusions

In conclusion, our study suggested that elevated SII levels are a significant and positive risk for ISR in diabetics. In addition, SII may be a useful marker for predicting ISR risk, particularly in patients with triple-coronary artery disease. Moreover, we discovered an almost linear correlation between SII and ISR in patients with diabetes, and higher SII levels are related to earlier ISR onset in diabetics. The accessibility and cost-effectiveness of SII measurements further enhance its clinical utility as a valuable tool for optimizing risk stratification and guiding interventions in ISR management. However, in-depth and well-designed research is needed to confirm these findings.

## Data Availability

The raw data supporting the conclusions of this article will be made available by the authors, without undue reservation.
